# Seropositivity for CMV and IL-6 levels are associated with grip strength and muscle size in the elderly

**DOI:** 10.1186/1742-4933-10-33

**Published:** 2013-08-13

**Authors:** Alixe HM Kilgour, Charlotte Firth, Rowan Harrison, Paul Moss, Mark E Bastin, Joanna M Wardlaw, Ian J Deary, John M Starr

**Affiliations:** 1 Centre for Cognitive Ageing and Cognitive Epidemiology, University of Edinburgh, 7 George Square, Edinburgh EH8 9JZ, UK; 2 Geriatric Medicine Unit, University of Edinburgh, Edinburgh, UK; 3 Brain Research Imaging Centre, Division of Neuroimaging Sciences, University of Edinburgh, Edinburgh, UK; 4 Department of Psychology, University of Edinburgh, Edinburgh, UK; 5 Clinical Research Imaging Centre, Queen’s Medical Research Institute, University of Edinburgh, Edinburgh, UK; 6 School for Cancer Sciences, University of Birmingham, Birmingham B15 2TT, UK

**Keywords:** Sarcopenia, Grip strength, Cytomegalovirus, Interleukin-6, Immunosenescence

## Abstract

**Background:**

Sarcopenia is an important cause of morbidity and mortality in older adults, with immunosenescence and inflammation being possible underlying mechanisms. We investigated the relationship between latent cytomegalovirus (CMV) infection, Interleukin 6 (IL-6) levels, muscle size and strength in a group of healthy older community-dwelling people.

**Methods:**

Participants were healthy volunteers from the Lothian Birth Cohort 1936 study. Participants had IL-6 level and CMV antibody titre measured at age 70 years and grip strength and a volumetric T1-weighted MRI brain scan (allowing measurement of neck muscle cross-sectional area (CSA)) at age 73. Markers of childhood deprivation were adjusted for in the analysis due to correlations between childhood deprivation and latent CMV infection.

**Results:**

866 participants were studied; 448 men (mean age 72.48 years, sd 0.70) and 418 women (mean age 72.51 years, sd 0.72). In men, CMV seropositivity was associated with smaller neck muscle CSA (p = 0.03, partial eta squared = 0.01), even after adjustment for IL-6 levels. Neck muscle CSA was not associated with CMV seropositivity in women, or CMV antibody titre or IL-6 level in either sex. Grip strength associated negatively with IL-6 level (right grip strength p<0.00001, partial eta squared 0.032 and left grip strength p<0.00001, partial eta squared 0.027) with or without adjustment for CMV serostatus or antibody titre. CMV status and antibody titre were not significantly associated with grip strength in either hand.

**Conclusion:**

These findings support the hypothesis that there is a relationship between markers of immunosenescence (i.e. CMV serostatus and IL6 level) and low muscle mass and strength and longitudinal studies in older cohorts are now required to investigate these relationships further.

## Introduction

Sarcopenia refers to the decline in both muscle mass and function that occurs with age [1-3]. It is an important cause of morbidity and mortality in older adults [4-6]. Longitudinal studies have shown muscle mass to be lost at approximately 1% per annum and strength to be lost at 2-4% per annum in older adults [7,8]. Despite its clinical importance, current understanding of the mechanisms underlying sarcopenia remains unclear. Immunosenescence and inflammation have both been identified as possible contributing factors. Immunosenescence is the age-associated impairment of immune function due to changes in both the innate and adaptive immune response [9]. These changes lead to a decreased ability to respond to pathogens, although an agreement on clinical biomarkers and associated clinical outcomes requires further research [9].

Seropositivity for cytomegalovirus (CMV, otherwise known as Human Herpes Virus 5), is common in older adults [10,11]. Most people are infected in childhood or young adulthood and become carriers of the virus in a latent state for the rest of their lives [12-14]. The role of CMV status in immunosenescence is a topic of current research, and it remains unclear whether the relationship is causal or associative. However, latent CMV infection has been linked to several clinical outcomes, including frailty and increased mortality. Several studies have found an association between CMV seropositivity and/or CMV antibody titre and the presence of atherosclerosis and coronary heart disease, with some studies also demonstrating a correlation with survival time [15-17]. Other studies have found associations between CMV and cognitive decline in older adults [18], and all cause mortality [19].

There is also evidence of an association between CMV infection and frailty. Aiello et al. [20] found that CMV antibody titre is negatively associated with the ability to carry out activities of daily living (ADLs) in elderly Latino subjects, after correcting for gender and age. However, this relationship became non-significant after adjusting for the total number of health conditions, body mass index, and household income. Schmaltz et al. found an association between frailty, defined using the Fried criteria, and CMV serostatus in older women [20]. Studies investigating frailty vary widely on the criteria used for diagnosis and not all frailty scores contain a measure of muscle mass [21,22]. Therefore, in order for clear conclusions to be drawn about the possible underlying mechanisms of sarcopenia, it is important to study it as an independent variable rather than as a component of a frailty score.

Interleukin 6 (IL-6) is a cytokine known to be part of the acute phase response, i.e. the initial immune system reaction to infection or trauma [23]. Increasing age is associated with latent low grade inflammation; levels of IL-6 appear to increase with age, particularly following the andropause or menopause [24,25]. In a large cross-sectional study of septuagenarians, raised IL-6 levels were associated with reduced muscle mass and strength [26]. However, in a further longitudinal cohort higher IL-6 levels were associated with loss of muscle strength, although no association was found with muscle mass [27].

Several studies investigating the effect of CMV on frailty and functional ability have adjusted for IL-6 to assess its role as a mediator, as CMV is known to increase IL-6 gene expression and production in peripheral blood mononuclear cells [28]. Indeed in the above mentioned study, Schmaltz et al. found that CMV positive subjects with high IL-6 levels had a significantly higher prevalence of frailty than those with a low IL-6 level [20]. Also, data from the Women’s Health and Aging Studies found that IL-6 appeared to modulate the effect of CMV antibody titre on frailty as an outcome (measured using the Fried criteria), although the effect did not reach statistical significance [29].

We could find no previous studies which have looked at the association between muscle mass and CMV serostatus or antibody titre. Furthermore we found only one study which addressed the relationship between CMV serostatus and handgrip strength [21], an important marker of muscle function in older age, and that study only looked at women. As detailed above, IL-6 may play an important mediatory role in these relationships and it is therefore important to study this in tandem with CMV status. In this study we investigated the relationship between latent CMV infection, IL-6 level and markers of sarcopenia (muscle size and strength) in a healthy older cohort of community-dwelling men and women.

## Results

There were 866 participants in wave 2 of the LBC1936 study; 448 men (mean age 72.48 years, sd 0.70) and 418 women (mean age 72.51 years, sd 0.72). This represents 79.4% of the participants who attended at the first wave of testing aged 70 years (n=1091). Baseline data for neck muscle CSA, right and left grip strength, CMV serostatus, CMV antibody titre and IL-6 levels are shown in Table 1. Baseline data for measures of childhood deprivation are shown in Table 2.

**Table 1:** Muscle size and strength, CMV and IL-6 baseline data

**Variable**	**Statistic/group**	**Men**	**Women**
**Total neck muscle CSA (mm**^**2**^**)**	Mean	2576.6	1814.5
	SD	421.0	281.2
	n	343	298
**Right grip strength (kg)**	Mean	35.49	21.28
	SD	6.82	5.54
	n	448	416
**Left grip strength (kg)**	Mean	34.69	19.93
	SD	6.57	5.13
	n	447	416
**CMV status**	Positive (%)	60.6	69.0
	Negative (%)	39.4	31.1
	n	439	409
**CMV antibody titre**	Median	60.56	132.11
	IQ range	0.47-214.00	2.40-277.78
	n	437	406
**IL-6 level**	Median	1.60	1.48
	IQ range	1.05-2.42	1.01-2.29
	n	425	390

**Table 2:** Childhood deprivation baseline data

**Variable**	**Statistic/group**	**Men**	**Women**
**Overcrowding index (age 11)**	Median	1.20	1.20
	IQ range	0.86-1.67	0.80-1.67
	n	447	416
**Indoor/outdoor toilet (age 11)**	Indoor (%)	87.5	90.0
	Outdoor (%)	12.5	10.0
	n	447	418
**Father’s social class**	I (%)	6.9	7.3
	II (%)	21.5	17.2
	III (%)	56.7	54.7
	IV (%)	6.9	13.3
	V (%)	7.9	7.6
	n	404	384
**Years spent in full time education**	Median	10	10
	IQ range	10-12	10-12
	n	448	418

We assessed associations between IL-6 and CMV antibody titre (using Spearman’s rho correlations) and CMV serostatus (using the Wilcoxon independent samples test) with age, height, weight, the muscle variables and the measures of childhood deprivation (Table 3). The association between CMV serostatus and indoor/outdoor toilet was analysed using the chi-square test. In men, being CMV seropositive or having a high CMV titre is associated with all the markers of childhood deprivation. In women, being CMV seropositive or having a high CMV titre is associated with a higher overcrowding index and lower social class of their father and in addition a high CMV titre is associated with fewer years of formal education. In men, IL-6 levels only significantly correlate with the number of years of full time education (i.e. the more years of education the lower the IL-6 level), whereas in women all the markers of childhood deprivation correlate with a higher IL-6 level, except for the indoor/outdoor toilet question. If IL-6 was acting as mediator for CMV infection (i.e. CMV infection causes inflammation, raising IL6 levels, which causes increased sarcopenia), we would expect a correlation between the two variables. However, the Spearman’s rho correlation between IL-6 and CMV antibody titre was non-significant (rho=0.06, p=0.07), while a Wilcoxon independent samples test for IL-6 and CMV serostatus was also non-significant (test statistic 1.28, p=0.20).

**Table 3:** Wilcoxon independent samples test (CMV serostatus) and Spearman’s rho correlations (CMV antibody titre and IL-6 level) (p values)

	**Male**			**Female**		
	**CMV status (pos/neg)**^**a**^	**CMV titre**^**b**^	**IL-6 titre**^**b**^	**CMV status (pos/neg)**^**a**^	**CMV titre**^**b**^	**IL-6 titre**^**b**^
**Age in days at wave 2**	.68	.09	.09	**2.32**	**.17**	.08
	*(.50)*	*(.06)*	*(.08)*	***(.02)***	***(<.01)***	*(.14)*
**Height in cm**	**−2.08**	**-.11**	**-.12**	**−2.50**	-.09	.00
	***(.04)***	***(.02)***	***(.01)***	***(.01)***	*(.07)*	*(.95)*
**Weight in kg**	1.44	.07	**.16**	−1.06	.02	**.30**
	*(.15)*	*(.15)*	***(<.01)***	*(.29)*	*(.71)*	***(<.001)***
**Total neck muscle CSA (mm**^**2**^**)**	−1.60	-.06	.09	1.02	.09	**.21**
	*(.11)*	*(.26)*	*(.10)*	*(.31)*	*(.12)*	***(<.001)***
**Grip strength right hand (kg)**	-.62	-.05	**-.25**	**−2.81**	**-.10**	-.07
	*(.54)*	*(.34)*	***(<.001)***	***(.01)***	***(.04)***	*(.16)*
**Grip strength left hand (kg)**	−1.95	**-.10**	**-.25**	**−2.04**	-.08	-.08
	*(.05)*	***(.03)***	***(<.001)***	***(.04)***	*(.12)*	*(.11)*
**Overcrowding index age 11**	**4.53**	**.17**	.03	**3.56**	**.16**	**.10**
	***(<.001)***	***(<.001)***	*(.56)*	***(<.001)***	***(<.01)***	***(.04)***
**Father’s job class as a number**	**3.82**	**.19**	.03	**2.51**	**.14**	**.16**
	***(<.001)***	***(<.001)***	*(.55)*	***(.01)***	***(<.01)***	***(<.01)***
**No. of years of full-time education**	**−3.07**	**-.17**	**-.13**	−1.57	**-.11**	**-.10**
	***(<.01)***	***(<.001)***	***(.01)***	*(.12)*	***(.01)***	***(.04)***
**Indoor=1 or outdoor=2 toilet at age 11**	0.47*	**.10**	.04	0.69*	.06	.05
	***(.02)***	***(.03)***	*(.37)*	*(.33)*	*(.18)*	*(.30)*

^a^Wilcoxon independent samples test statistic (and associated p values) for CMV status and all predictor variables except indoor/outdoor toilet age 11, which was analysed using the chi-square test and shows *the odds ratio for being CMV seropositive if indoor toilet age 11.

^b^The columns for CMV titre and IL-6 titre show the Spearman’s rho correlation with the predictor variables (and the associated p value).

General linear models (ANCOVAs) were then created for each muscle variable separately with each measure of immune status (ie CMV status, CMV titre and IL-6 level) before rerunning the models of CMV status and antibody titre adjusting for IL-6 status also. Tables 4 and 5 contain the results for the ANCOVA for CMV serostatus and neck muscle CSA, and IL-6 and grip strength. The p value gives the significance of the independent variable’s association and the partial eta squared gives a measure of effect size, for which Cohen [30] says the following: small ≥0.0099; medium ≥0.0588; large ≥0.1379.

**Table 4:** ANCOVA for CMV status and total neck muscle CSA

**Source**	**Sig.**	**Partial eta squared**
**Age in days at wave 2**	.38	<.01
**Weight in kg**	<.001	.22
**Sex (Male=1, Female=2)**	<.001	.42
**Overcrowding index age 11**	.29	<.01
**Indoor=1 or outdoor=2 toilet at age 11**	.88	<.01
**Father’s job class as a number**	.70	<.01
**No. of years of full-time education**	.43	<.01
**CMV serostatus (neg=1, pos=2)**	.10	.01
**Sex by CMV serostatus**	.028	.01

**Table 5:** ANCOVA for IL-6 titre and grip strength right and left hands

		**Right hand**		**Left hand**
**Source**	**Sig.**	**Partial eta squared**	**Sig.**	**Partial eta squared**
**Age in days at Wave 2**	.005	.01	.004	.01
**Height**	<.001	.08	<.001	.07
**Sex (Male=1, Female=2)**	<.001	.28	<.001	.34
**Overcrowding index age 11**	.72	.00	.25	.00
**Indoor=1 or outdoor=2 toilet at age 11**	.75	.00	.64	.00
**Father’s job class as a number**	.95	.00	.65	.00
**No. of years of full-time education**	.051	.01	.10	.00
**IL-6 Level**	<.001	.03	<.001	.03

We found that CMV seropositivity was associated with a smaller neck muscle CSA in men but not in women (p = 0.028, partial eta squared = 0.01) (Table 4). In this model, lower weight and female sex were also associated with smaller neck muscle CSA. When this model was corrected for IL-6 level the effect remained significant (p = 0.047). The model for neck muscle CSA and CMV antibody titre showed no significant association, both with and without adjustment for IL-6 level, nor did the model with IL-6 as predictor variable, without adjusting for CMV infection.

Weaker grip strength in both right and left hands was found to be associated with higher IL-6 level; right grip strength p<0.00001, partial eta squared = 0.032 and left grip strength p<0.00001, partial eta squared = 0.027 (Table 5). The associations remain strongly positive even after adjustment for CMV status (p<0.0001) and CMV antibody titre (p<0.0001). In these models (shown in Table 5), older age, shorter stature and female sex were also significantly associated with weaker grip strength in both hands. The models using CMV status and antibody titre alone were not significantly associated with grip strength in either hand.

## Discussion

This report used data from waves 1 and 2 of a population-based elderly cohort study, to investigate the relationship between latent CMV infection, IL-6 levels and sarcopenia, measured using neck muscle CSA and grip strength in both hands. There was no significant group difference for sex, age or CMV status and titre between those who participated in wave 1 but not wave 2 (n=225) and those who participated in both waves (n=866) (independent t tests, p>0.05). We found that men who were seropositive for CMV antibody at age 70 years had a neck muscle CSA on average 4% smaller at age 73 than men who were seronegative. This effect remained positive whether adjusting for IL-6 level or not. It is well documented that muscle mass is lost at roughly 1% per year [7,8], therefore being a man who is CMV seropositive in your 70 s confers the same risks of low muscle bulk as being 4 years older. We did not detect a significant association in women between CMV serostatus and neck muscle CSA, or between CMV serostatus and grip strength in either hand. Other studies have postulated that as CMV seropositivity is so common in older adults it may be more important to measure CMV antibody titre itself. However, we found no association between CMV antibody titre and either neck muscle CSA or grip strength. This result may indicate that latent CMV infection leads to increased muscle loss over an extended period, as CMV is commonly acquired in childhood, though there is the possibility of temporary reactivations of CMV throughout life, and that the titre reflects the current situation, which may have less impact on muscle bulk. Longitudinal studies will be able to explore these relationships further.

IL-6 levels were found to strongly predict grip strength in both right and left hands in men and women. IL-6 predicted 3.2% of the variance in right-sided grip strength and 2.7% of the variance in left-sided grip strength. These associations remained significant when adjusting for CMV serostatus or antibody titre. We found no significant association between IL-6 levels and neck muscle CSA. Therefore our findings do not support previous work that has found that IL-6 may act as a mediator by which latent CMV infection causes frailty [20,29].

It is widely accepted that muscle size and strength do not decline in a parallel manner [31,32], therefore they are not purely a function of each other and it may be that different factors cause decline in one parameter more than the other, as our results have shown. Similarly, a study looking at the effect of IL-6 levels on muscle found a significant association with decline in grip strength but not muscle mass [27], again indicating parameter-specific effects. Also, these results are based on cross-sectional data and therefore do not necessarily reflect changes with age. Therefore it could be that individuals with latent CMV infection or lifelong raised IL6 levels have always had smaller/weaker muscles, rather than an increased rate of decline in muscle mass or function with age. However as sarcopenia is currently diagnosed using reference to peers or a healthy young population, having a lower peak muscle mass and function should still be considered risk factors for sarcopenia. Longitudinal studies may help elucidate these relationships further.

The sole previous study we found that investigated the relationship between CMV status and grip strength only included women [21]. They found no significant difference in grip strength between seropositive and seronegative women. We have replicated this finding, but found a sex specific effect between male sex and CMV serostatus. There is evidence that men lose more muscle mass than women with age even after correction for body stature [33], therefore differing factors may play more or less of a role between the genders. Additionally, their muscles are larger to start with and therefore a reduction may be easier to detect.

It is unclear how CMV might directly influence physical functioning, however latent CMV infection has been identified as an important component of an immune risk phenotype that is associated with immunosenescence, inflammation, and several latent health conditions observed with aging [34]. All these may represent possible contributory mechanisms to sarcopenia. For example, if CMV infection predisposes to cardiovascular disease this, in turn, might limit exercise and hence loss of muscle strength and size. Should our findings be confirmed, exploration of potential causal pathways will be warranted.

Raised plasma IL-6 levels are known to increase proteolysis within muscle, by upregulating the proteolytic UPP pathway [35], however it is not known if the degree of increase seen with ageing is enough to cause atrophy and it is thought that proteolysis is not a major factor in normal ageing muscle [36]. However, IL-6 may exert its effect through less direct routes. It is known that IL-6 causes anorexia, which would lead to decreased protein substrate. Also, IL-6 can both activate cortisol secretion and induce 11beta-hydroxysteroid type 1 expression, so it may exert its effect via the steroid pathway [37,38]. Furthermore, animal studies have demonstrated that inflammatory cytokines can induce muscle apoptosis by DNA fragmentation [35], though such models may not represent low level inflammatory changes occurring over a prolonged period.

The narrow geographic, age and ethnic mix within the LBC1936 cohort means this study may not prove generalisable. However, the narrow age range helps to reduce the powerful effect of advancing age on many of the parameters measured in this and other studies and which may have lead to the impression of stronger direct relationships between co-associated variables than is actually the case. Additionally the size of the sample studied and the fact that we replicated results in some of our analyses found in other work is reassuring. The high correlation between the markers of childhood deprivation and CMV status were as predicted and raise the possibility that other correlates of childhood socioeconomic deprivation may be mediated by CMV infection, although this relationship may be less strong in other populations. Also, when studying sarcopenia it is important to consider rate of decline rather than solely cross-sectional measures. Therefore in the future, longitudinal studies will be crucial in developing an understanding of these relationships. Finally, the concept of a homogeneous model of sarcopenia, whereby all muscle throughout the body ages at the same rate, is proving increasingly unlikely to be valid with studies showing rates of muscle ageing to vary around the body [33,39,40]. Therefore whilst our measure of neck muscle CSA has previously been shown to correlate strongly to mid-thigh muscle CSA, it is important to consider that different factors may worsen or ameliorate muscle ageing in different muscle groups throughout the body.

## Conclusion

In a large population-based elderly cohort study we found the men who were seropositive for CMV had smaller neck muscle CSA than men who were seronegative, and this effect was independent of IL-6 level. We also found that higher IL-6 levels, but not CMV levels, were strongly associated with lower grip strength in both hands in men and women. These associations were not attenuated when the model was adjusted for CMV serostatus or antibody titre. These findings support the hypothesis that there is a relationship between immunosenescence and markers of sarcopenia and longitudinal studies are now required to investigate these relationships further.

## Methods

### Participants – The Lothian Birth Cohort 1936 (LBC1936)

The LBC1936 study consists of 1091 relatively healthy, age-homogeneous older people who, at the age of 11 years, participated in the Scottish Mental Survey of 1947, when they sat a general mental ability test, the Moray House Test number 12 (MHT). The cohort, including the imaging protocol, has been described previously in detail [41,42] At age 70 years they underwent a series of cognitive tests (including retaking the MHT), and physical and biochemical tests, at the Wellcome Trust Clinical Research Facility (WTCRF) at the Western General Hospital, Edinburgh, where 866 returned for further testing at age 73. All participants gave written, informed consent to the study. All participants were Caucasian and almost all lived independently in the Lothian region (Edinburgh city and surrounding area) of Scotland.

### Neck muscle cross-sectional area

In this study we use neck muscle cross-sectional area (CSA) as a validated measure of muscle size [32]. We have previously shown in a study of 24 subjects that neck muscle CSA is strongly correlated with thigh muscle CSA (R^2^ = 0.77), which is often used as a proxy for general muscle bulk. Neck muscle CSA is generally available in brain MRI studies, whereas thigh muscle CSA is not usually measured within longitudinal cognitive ageing studies.

Participants underwent a volumetric brain MRI scan as part of the LBC1936 study. MRI was performed with participants in a supine position using a 1.5 T clinical scanner (Signa HDxt, GE Healthcare, Milwaukee, USA) at the Brain Research Imaging Centre, University of Edinburgh (http://www.bric.ed.ac.uk). A phased array eight channel head coil was used and inversion recovery prepared volumetric T1 weighted images were acquired in a coronal plane for each participant; the scan alignment was perpendicular to the long axis of the hippocampus determined from a preliminary T2-weighted sagittal sequence. The flip angle was 8°, bandwidth 15.63 KHz, echo time (TE) 4 to 13 ms, repetition time (TR) 9.6 ms and inversion or preparation time (TI) 500 ms. The field of view (FOV), fixed superiorly at the cranial vertex, was 25.6 × 25.6 cm, the acquisition matrix was 192×192, with 160 slices acquired with a slice thickness of 1.3 mm with no slice gap. These data took 8.13 minutes to acquire per patient.

Neck muscle CSA was measured using a validated technique as described previously [43]. In summary, the mid-point of the C2-vertebra was located in the sagittal slice of a 3D reconstructed image. The image was then converted to a transverse view and the posterior neck muscles were outlined using a cursor on a dedicated workstation. The software then calculated the contained area. The muscles groups measured were the semispinalis capitis, splenius capitis and trapezius (measured as a combined group), and the sternocleidomastoid. Each measurement was performed three times and the median for each value was used for the following analyses.

### Grip strength

Grip strength was measured with a Jamar Hydraulic Hand Dynamometer, with all participants performing 3 trials with their right and left hands; the best of the 3 trials was used for the following analyses.

### CMV and IL-6 measures

CMV was measured in plasma samples collected at age 70, using a CMV ELISA assay. Mock and viral-infected lysate was coated onto ELISA plates and incubated overnight. Standards (a mixture of three CMV positive plasma samples) and plasma samples were added to the plates and incubated for one hour before washing. An anti-IgG horseradish peroxidase conjugated secondary antibody was then added to the plate to incubate for one hour. After washing, TMB substrate was added and the reaction stopped by addition of 1M HCL. The sample was assessed using an ELISA reader at 450 nm. To determine CMV titres, mock values were first subtracted from lysate values. The data were then analysed in PRISM, and CMV titres were calculated with reference to the standard curve. Values above 10 were considered to be seropositive. To ensure accuracy, all samples were tested in duplicate. IL-6 levels were analysed at the University of Glasgow using high sensitivity ELISA from R&D Systems. The minimum detectable dose ranged from 0.016-0.110 pg/mL (mean=0.039 pg/mL). The intra-assay CV ranged from 6.9 to 7.8%, while the inter-assay coefficient of variance ranged from 6.6 to 9.6%.

### Childhood deprivation

At the age 70 assessment, participants were asked to provide background demographic and environmental information about their childhood, specifically for when they were aged about 11 years. Participants reported the number of people they lived with and the number of rooms in the house, which was used to calculate an overcrowding index (people/room). Participants also reported: whether their household had indoor or outdoor toilet facilities; their father’s occupation to allow father’s social class to be coded (categorised from I, professional, to V, unskilled); and the number of years they spent in full-time, formal education.

### Statistical analysis

Descriptive statistics, exploratory analyses and general linear modeling (Analysis of Covariance; ANCOVA) were performed using SPSS version 18.0 for Windows (SPSS Inc, Chicago, Ill, USA). Missing values were excluded listwise for the ANCOVA analyses. For the ANCOVA, we constructed baseline models with the measures of neck muscle CSA and grip strength (right and left) as dependent (i.e. outcome) variables and CMV serostatus, CMV antibody titre and IL-6 level as independent variables, adjusting for age, gender and either height or weight, as a measure of body size, and the four measures of childhood deprivation. Total neck muscle CSA was found to correlate more strongly with weight (P<0.001), whereas both right and left grip strength correlated more strongly with height (p<0.001). Therefore we used the respective measures for adjustment in each of the analyses.

## Competing interests

The authors declared that they have no competing interests.

## Authors’ contributions

AHMK and JMS proposed the hypotheses, performed the analyses and drafted the manuscript. AHMK performed the neck cross-sectional area measurements. IJD, JMS, MEB and JMW designed the LBC1936 study. CF, RH and PM designed and implemented the immunological measurements. All authors approved the final manuscript.
